# Loneliness and its impact on quality of life in Chinese heroin-dependent patients receiving methadone maintenance treatment

**DOI:** 10.18632/oncotarget.19565

**Published:** 2017-07-26

**Authors:** Ying-Jia Yang, Yan-Min Xu, Wen-Cai Chen, Jun-Hong Zhu, Jin Lu, Bao-Liang Zhong

**Affiliations:** ^1^ Shenzhen Key Laboratory for Psychological Healthcare, Shenzhen Institute of Mental Health, Shenzhen Kangning Hospital, Shenzhen Mental Health Center, Shenzhen, Guangdong Province, China; ^2^ Affiliated Wuhan Mental Health Center (The Ninth Clinical School), Tongji Medical College of Huazhong University of Science and Technology, Wuhan, Hubei Province, China; ^3^ Department of Psychiatry, The First Affiliated Hospital of Kunming Medical University, Kunming, Yunnan Province, China

**Keywords:** heroin dependence, methadone, loneliness, quality of life

## Abstract

To date, no studies have examined loneliness in Chinese patients with substance use disorders. This study determined the prevalence and socio-demographic and clinical correlates of loneliness and its impact on quality of life (QOL) in Chinese heroin-dependent patients (HDPs) receiving methadone maintenance treatment (MMT). A total of 603 HDPs were consecutively recruited from three city-owned MMT clinics in Wuhan, China, and administered with a standardized questionnaire to collect socio-demographic and clinical data. Loneliness and QOL were assessed with a single-item self-report question and World Health Organization QOL Scale Brief Version, respectively. As high as 55.9% Chinese HDPs of MMT clinics endorsed loneliness. Multiple logistic regression found that non-married status, unemployment, religious beliefs, a history of injecting heroin, poor interpersonal relationship, and more depressive symptoms were significant contributors to loneliness. Lonely HDPs had significantly poorer physical and psychological QOL than not lonely HDPs. After controlling for the potential confounding effects of socio-demographic and clinical factors with analysis of covariance, these group-differences in physical (F = 127.169, *P* < 0.001) and psychological (F = 85.004, *P* < 0.001) QOL remained statistically significant. Loneliness is prevalent in HDPs receiving MMT and independently associated with poor QOL. To address this serious issue, psychosocial services, including the identification of psychosocial problems, expanded social supports that focus on promoting mental wellbeing, and, when necessary, psychiatric assessment and treatment, should be routinely provided in Chinese MMT settings.

## INTRODUCTION

Loneliness is significant in the course of substance dependence: as a cause for addiction, a maintaining factor for repeated substance use, and a trigger for addiction relapse [1–4]. For example, people may use drugs and alcohol to cope with negative feelings such as depression and loneliness, and recovered addicts may turn to substances again if they fail to overcome the loneliness of their life after recovery. In addition, there is evidence that addiction could lead to isolation and loneliness, because individuals who are lost to an active addiction unconsciously tend to alienate friends and damage relationships with family members [1, 3, 5]. Importantly, repairing a broken relationship can be very difficult for addicts during or after the recovery, leading to increased risk of relapse via the mechanisms of loneliness. Therefore, there is a vicious cycle between loneliness and addiction, and taking measures to relieve loneliness is very promising in the prevention and management of addiction.

In contemporary China, methadone maintenance treatment (MMT) has been a national strategy to reduce the harms of illegal drug (mainly heroin) dependence [6]. Studies have shown that MMT is effective in reducing drug-related crimes, HIV risk behaviors, and incidences of HIV and HCV, and improving the health and social function of Chinese individuals with heroin dependence [7–11]. China’s MMT program was initiated in 2004, rapidly expanded since then, and, until now, China has built the world’s largest MMT network [12, 13]. However, there still are many challenges in providing services in Chinese MMT clinics. Due to the lack of mental health professionals and social workers, health services provided in most MMT clinics in China are MMT only. Even in some MMT clinics managed by psychiatric hospitals, psychiatrists treating patients dependent on heroin and other illicit drugs are largely unaware of the importance of the recognition and management of psychosocial problems [14]. This seriously undermines the effectiveness of the clinical treatments provided by Chinese MMT clinics.

Quality of life (QOL) is an important outcome measure of chronic disease management and treatment, including drug-addiction maintenance treatment programs [15, 16]. Studies assessing the QOL of Chinese heroin-dependent patients (HDPs) in MMT clinics have observed a significantly improved QOL after entering the MMT but still reported a significantly poorer QOL compared to healthy controls [15, 17, 18]. A number of factors, including socio-demographic variables, heroin use characteristics, family support, depression, and pain are related to poor QOL in Chinese HDPs [17, 19, 20]. Until now, however, the impact of loneliness on QOL of HDPs has not been investigated, although the significant effect of loneliness on QOL of the elderly population has been reported [21].

Prior studies have linked loneliness to a range of physical and mental health problems, including high blood pressure, increased mortality, daytime dysfunction, cognitive impairment, depression and suicidal behaviors [22–26]. In Chinese society, due to the lack of public awareness of heroin dependence and the stigma associated with addiction, HDPs in and after recovery are generally excluded from the job market, leading to their difficulties in returning to society and normal life [27]. Owing to such social exclusion, Chinese HDPs under MMT may experience a high level of loneliness. To date, however, no study of loneliness in Chinese HDPs has been undertaken in China.

Given that little is known on characteristics of loneliness in China’s addiction treatment practice, this study was set out to investigate the prevalence and socio-demographic and clinical correlates of loneliness and its impact on QOL in Chinese HDPs from MMT clinics.

## RESULTS

A total 652 eligible patients were enrolled in this study, 33 did not complete the questionnaire, and 16 refused to participate; leaving 603 (92.5%) completed the survey. The average age of the 603 subjects was 38.1 years (standard deviation [SD] = 7.0, range = 21–59), and 68.3% were women. Most patients (84.1%) injected heroin before being admitted to MMT clinics, and the mean dose and duration of methadone were 73.1 mg/d (SD = 57.6) and 24.8 months (SD = 11.7), respectively. The 603 completers and 49 non-completers were comparable in proportion of males (31.7% v. 28.6%, χ^2^ = 0.202, *P* = 0.653) and age (38.1 ± 7.0 v. 36.5 ± 6.8, t = 1.542, *P* = 0.124).

In total, 337 HDPs endorsed loneliness. The prevalence of loneliness was 55.9% in the whole sample, with 59.5% in males and 48.2% in females. Results of the comparison between not lonely and lonely groups (Table 1) showed that, compared to not lonely HDPs, lonely HDPs were significantly more likely to be non-married, be unemployed, have religious beliefs, get poor along with others, have a history of injecting heroin, take methadone for a longer period, and have more depressive symptoms.

**Table 1:** Characteristics and quality of life (QOL) of heroin-dependent patients with and without loneliness

Characteristics	No loneliness group (*N* = 266)	Loneliness group (*N* = 337)	Statistics	*P*
Gender: male	167(62.8)	245(72.7)	χ^2^ = 2.951	0.086
Age (years)	37.6(7.6)	38.5(6.4)	t = 1.511	0.132
Education years	9.7(2.5)	9.4(2.5)	t = 0.992	0.322
Marital status: non-married*	101(38.0)	207(61.4)	χ^2^ = 21.463	< 0.001
Unemployment	125(47.0)	207(61.4)	χ^2^ = 8.288	0.004
Religious beliefs	25(9.4)	61(18.1)	χ^2^ = 13.068	< 0.001
Poor interpersonal relationship	167(62.8)	263(78.0)	χ^2^ = 16.203	< 0.001
History of injecting heroin	211(79.3)	296(87.8)	χ^2^ = 6.891	0.009
Duration of heroin use (years)	9.7(4.1)	10.1(4.3)	t = 0.978	0.329
Methadone dosage (mg/day)	70.0(28.9)	69.1(30.5)	t = 0.343	0.732
Duration of MMT	23.1(10.4)	25.7(11.1)	t = 2.843	0.005
SDS	37.3(7.5)	39.6(9.0)	t = 3.166	0.002
Physical QOL	25.6(3.7)	20.8(4.3)	t = 13.929	< 0.001
Mental QOL	19.7(3.0)	16.8(3.4)	t = 10.739	< 0.001

*Non-married included never-married, remarried, cohabitating, separated/divorced, and widowed.

Multiple logistic regression (Table 2) revealed that non-married status, unemployment, having religious beliefs, poor interpersonal relationships, injecting heroin, and more depressive symptoms were significantly associated with loneliness.

**Table 2:** Multiple binary logistic regression on correlates of loneliness in heroin-dependent patients

Factor	Risk level	Reference level	Coefficient	Standard error	Wald χ^2^	*P*	OR(95%CI)
Marital status	Non-married*	Married	0.793	0.197	16.165	< 0.001	2.21(1.50,3.26)
Unemployment	Yes	No	0.396	0.196	4.109	0.043	1.49(1.01,2.18)
Religious beliefs	Yes	No	0.817	0.316	6.678	0.01	2.26(1.22,4.21)
Interpersonal relationship	Poor	Good	0.878	0.217	16.293	< 0.001	2.41(1.57,3.68)
Usual route of heroin administration	Injecting	Smoking	0.562	0.252	4.978	0.026	1.75(1.07,2.87)
SDS	Low	High	0.041	0.012	10.513	0.001	1.04(1.02,1.07)

*Non-married included never-married, remarried, cohabitating, separated/divorced, and widowed.

Lonely HDPs had significant poorer physical and psychological QOL than not lonely HDPs (Table 1). After controlling for the potential confounding effects of socio-demographic and clinical factors with analysis of covariance (ANCOVA), differences between lonely and not lonely HDPs remained statistically significant in both physical (F = 127.169, *P* < 0.001) and psychological (F = 85.004, *P* < 0.001) health domains of QOL.

## DISCUSSION

To the best of our knowledge, this is the first study in China that examined the characteristics of loneliness in patients with substance use disorders, as well as its negative effect on the QOL of these patients. We found that 55.9% Chinese HDPs receiving MMT felt lonely at least sometimes. Compared to studies using similar measures and definitions of loneliness, this prevalence is higher than that reported among adult New Zealanders (15.7%) [28], Danish older adults in general practice (17.9%) [29], Swedish community older adults (17.2%) [30], Chinese migrant workers (18.3%) [11], Chinese community older adults (33%) [22], Finnish community-dwelling older adults (39.4%) [31], and Portuguese community-dwelling adults (38%) [32]. Although direct comparisons between our study and these earlier studies are potentially problematic due to variations in populations (i.e., HDPs vs. older adults), methods of data collection (i.e., self-report vs. interview), sampling methods (i.e., random vs. convenient), and study settings (i.e., clinics vs. communities), our finding that over a half of HDPs experienced loneliness clearly indicates that loneliness is very common in HDPs of MMT clinics in China.

There is convincing evidence that socially isolated individuals are at greater risk for experiencing loneliness [10, 22]. Because individuals who are unmarried or have poor relationships with others are more likely to be socially isolated, we found non-married status and poor interpersonal relationships to be significant risk factors of loneliness in HDPs. Previous studies rarely reported the significant association between unemployment and loneliness. The significant unemployment-loneliness link in HDPs may be ascribed to the small social network of unemployed individuals, because the job provides a good opportunity to connect with others such as colleagues and clients.

It is unusual to find a significant relationship between religious beliefs and loneliness in HDPs, because holding a religious belief is a protective factor against negative emotion in Western literature [33, 34]. One reasonable explanation is that most Chinese are atheists; persons who subsequently become religious may do so as they are emotionally troubled by loneliness.

In this study, a history of injecting heroin was significantly associated with loneliness of HDPs. In theory, injecting heroin could not directly result in feeling of loneliness. In general, HDPs with a history of injecting heroin should have more infectious diseases (i.e., HBV, HCV, and HIV) and painful medical conditions (i.e., skin infection caused by needle-sharing), and physical disabilities due to these conditions would further limit individuals’ ability to take part in social activities, which in turn cause loneliness [22], therefore, loneliness may be an indirect result of injection heroin in HDPs.

In line with earlier findings [25, 35, 36], depressed HDPs were more likely to be lonely. This may be explained by the fact that there is a reciprocal relationship between depressive symptoms and loneliness, that is, depression increases the risk of developing loneliness and vice versa.

Because loneliness is significantly associated with physical and mental morbidities [22–26], the poor physical and psychological QOL among lonely HDPs was expected. In addition, loneliness can influence one’s perceptions about health [37] and QOL is a subjective measure of overall health [15], the negative perception of lonely HDPs can also explain their poor QOL.

These results should be interpreted with caution due to the following methodological limitations. First, the results are applicable only to community-dwelling HDPs receiving MMT. In addition, the study sample was recruited from MMT clinics in Wuhan, China, so the findings may not be generalized to HDPs of other parts of China. Second, the study was cross-sectional in nature, therefore the causality of relationship between loneliness and socio-demographic and clinical variables could not be explored. Due to the same reason, the significant impact of loneliness on QOL is still questionable and needs to be validated in prospective studies. Third, information on the utilization of psychosocial services of lonely HDPs is important for policy-making for reducing loneliness at MMT clinics. However, due to limitation in the study design, this study did not collect such data. The final limitation is that some other risk factors of loneliness such as medical conditions were not assessed in the study.

In summary, the present study has demonstrated a high prevalence of loneliness in Chinese HDPs receiving MMT, and loneliness may impair their QOL. There is an urgent need for health workers of Chinese MMT clinics to address the epidemic of loneliness in HDPs. Efforts to reduce loneliness of HDPs may be useful to target on those who are unmarried, unemployed, and depressed, and have religious beliefs, get poor with others, and have a history of injecting heroin. Psychosocial and medical services for HDPs in MMT clinics should include periodical assessment of loneliness, expanded social supports for promoting mental wellbeing, and appropriate psychiatric treatment when necessary.

## MATERIALS AND METHODS

### Subjects

This cross-sectional survey was conducted in three city-owned MMT clinics in Wuhan, Hubei Province, China. Wuhan is the most populous city in Central China with a population of 10.6 million persons in 2015. According to official statistics in 2015, the number of illicit drug users in this city was estimated to be 0.7 million. HDPs, who were aged 20 years or older, met DSM-IV diagnostic criteria for lifetime heroin dependence, and taking methadone in these clinics, were consecutively recruited during the study period. Patients with severe physical illnesses, alcohol dependence, brain organic mental disorders, or psychotic symptoms were excluded.

The Institutional Review Board of Wuhan Mental Health Center approved the research protocol. Written informed consent was obtained from all participants.

### Assessments and procedures

Before the formal study, a pilot study was performed to test the feasibility of our study procedures. The survey questionnaire was also finalized after the pilot testing.

We used the questionnaire to collect socio-demographic and clinical data, including gender, age, education years, marital status, employment, self-reported presence of religious beliefs, interpersonal relationship, usual route of heroin administration (injection or smoking), duration of heroin use, duration of MMT, methadone dosage, and Zung’s Self-rating Depression Scale (SDS) ^40, 41^. In this questionnaire, all respondents were asked “Do you have any religious beliefs?” Patients answering “yes’’ were classified as having religious beliefs. Interpersonal relationship was assessed by asking the respondent how he/she got along with others on a 3-point scale: 1 = poor, 2 = fair, 3 = well. The Chinese SDS is a 20-item self-report scale to assess the severity of depressive symptoms using a 4-point rating scale (1 = a little of the time to 4 = most of the time) ^40^. Total SDS score varies between 20 and 80, with higher scores denoting more depressive symptoms.

Loneliness was assessed with a single question asking how often the respondent feels lonely on a 5-point Likert scale: 1 = always, 2 = often, 3 = sometimes, 4 = seldom, 5 = never. This single-item measure of loneliness was widely used in prior studies [22, 30, 31, 38] and is well accepted by illiterate individuals [22, 38]. However, due to the stigma associated with loneliness, this direct measure may lead to an underestimate of the severity of loneliness [38]. Therefore, in accordance with previous studies [10, 30, 31], patients were classified as lonely if they indicated their loneliness was “sometimes”, “often”, or “always”, while patients reported “never” or “seldom” were classified as not lonely.

QOL was assessed with the Chinese version of the World Health Organization QOL Scale Brief Version (WHOQOL-BREF) [15, 39]. This scale comprises 26 items, consisting of four domains: physical health, psychological health, social relationships, and environment. Each item is rated on a 5-point Likert scale ranging from 1 (‘‘not at all’’) to 5 (‘‘extremely’’). Each domain is scaled in a positive direction with higher scores indicating a better QOL. The Chinese WHOQOL-BREF has been proved to be reliable and valid in HDPs under MMT [15]. To minimize the survey burden on subjects, only items of the physical and psychological QOL domains were used in this study.

The study investigators were trained young psychiatrists, who were also staff of these MMT clinics. They were arranged to give instructions for filling out the questionnaires and read out questions for subjects who were illiterate or had difficulties in reading.

### Statistical analysis

Prevalence of loneliness was calculated. Socio-demographic and clinical characteristics and QOL of lonely and not lonely groups were described and compared by *t*-test or Chi-square test, as appropriate. Multivariable logistic regression model with a backward stepwise entry of significant variables in the above univariate analysis was used to identify factors significantly associated with loneliness. Odds ratios (ORs) and 95% confidence intervals (CIs) were used to quantify the associations between factors and loneliness. To explore the independent association of QOL with loneliness, ANCOVA was used to adjust for potential confounding effects of socio-demographic and clinical variables. The statistical significance level was set at *p* < 0.05 (two-sided). SPSS software version 20.0 package was used for analyses.

## References

[1] Segrin C, McNelis M, Pavlich CA (2017). Indirect Effects of Loneliness on Substance Use through Stress. Health Commun.

[2] Mannes ZL, Burrell LE, Bryant VE, Dunne EM, Hearn LE, Whitehead NE (2016). Loneliness and substance use: the influence of gender among HIV+ Black/African American adults 50+. AIDS Care.

[3] Akerlind I, Hornquist JO (1992). Loneliness and alcohol abuse: a review of evidences of an interplay. Soc Sci Med.

[4] Blum K, Thompson B, Demotrovics Z, Femino J, Giordano J, Oscar-Berman M, Teitelbaum S, Smith DE, Roy AK, Agan G, Fratantonio J, Badgaiyan RD, Gold MS (2015). The Molecular Neurobiology of Twelve Steps Program & Fellowship: Connecting the Dots for Recovery. J Reward Defic Syndr.

[5] Hosseinbor M, Yassini Ardekani SM, Bakhshani S, Bakhshani S (2014). Emotional and social loneliness in individuals with and without substance dependence disorder. Int J High Risk Behav Addict.

[6] Sullivan SG, Wu Z, Rou K, Pang L, Luo W, Wang C, Cao X, Yin W, Liu E, Mi G (2015). National Methadone Maintenance Treatment Working G. Who uses methadone services in China? Monitoring the world’s largest methadone programme. Addiction.

[7] Zou X, Ling L, Zhang L (2015). Trends and risk factors for HIV, HCV and syphilis seroconversion among drug users in a methadone maintenance treatment programme in China: a 7-year retrospective cohort study. BMJ Open.

[8] Wu F, Peng CY, Jiang H, Zhang R, Zhao M, Li J, Hser YI (2013). Methadone maintenance treatment in China: perceived challenges from the perspectives of service providers and patients. J Public Health (Oxf).

[9] Zhong BL, Chiu HF, Conwell Y (2016). Rates and characteristics of elderly suicide in China, 2013–14. J Affect Disord.

[10] Zhong BL, Chen SL, Conwell Y (2016). Effects of Transient Versus Chronic Loneliness on Cognitive Function in Older Adults: Findings From the Chinese Longitudinal Healthy Longevity Survey. Am J Geriatr Psychiatry.

[11] Zhong B, Xu Y, Jin D, Zou X, Liu T (2016). Prevalence and correlates of loneliness among Chinese service industry migrant workers: A cross-sectional survey. Medicine.

[12] Shen J, Wang M, Wang X, Zhang G, Guo J, Li X, Li J (2016). Predictors of Poor Adherence to Methadone Maintenance Treatment in Yunnan Province, China. J Addict Med.

[13] Yang M, Zhou L, Hao W, Xiao SY (2014). Drug policy in China: progress and challenges. Lancet.

[14] Zhong B, Xiang Y, Cao X, Li Y, Zhu J, Chiu HF (2014). Prevalence of antisocial personality disorder among Chinese individuals receiving treatment for heroin dependence: a meta-analysis. Shanghai Arch Psychiatry.

[15] Zhu JJ, Wang Y, Zhong BL (2011). Reliability and validity of Chinese eition of the World Health Organization’s Quality of Life Questionnaire-Brief Version for outpatients on methadone maintenance treatment. Chin J Drug Depend.

[16] Vanagas G, Padaiga Z, Subata E (2004). Drug addiction maintenance treatment and quality of life measurements. Medicina (Kaunas).

[17] Xu YM, Zhong BL, Zhu JH, Liu TB (2014). Quality of life and its associated factors of heroin dependent patients receiving methadone -maintainance treatment. Chin J Drug Depend.

[18] Xiao L, Wu Z, Luo W, Wei X (2010). Quality of life of outpatients in methadone maintenance treatment clinics. J Acquir Immune Defic Syndr.

[19] Liu Y, Zhong BL, Zhu JH (2017). Pain and its association with quality of life in heroin-dependent patients receiving methadone maintenance treatment. Chin J Pain Med.

[20] Yen CN, Wang CS, Wang TY, Chen HF, Chang HC (2011). Quality of life and its correlates among heroin users in Taiwan. Kaohsiung J Med Sci.

[21] Arslantas H, Adana F, Abacigil Ergin F, Kayar D, Acar G (2015). Loneliness in Elderly People, Associated Factors and Its Correlation with Quality of Life: A Field Study from Western Turkey. Iran J Public Health.

[22] Zhong BL, Chen SL, Tu X, Conwell Y (2017). Loneliness and Cognitive Function in Older Adults: Findings From the Chinese Longitudinal Healthy Longevity Survey. J Gerontol B Psychol Sci Soc Sci.

[23] Luo Y, Hawkley LC, Waite LJ, Cacioppo JT (2012). Loneliness, health, and mortality in old age: a national longitudinal study. Soc Sci Med.

[24] Hawkley LC, Thisted RA, Masi CM, Cacioppo JT (2010). Loneliness predicts increased blood pressure: 5-year cross-lagged analyses in middle-aged and older adults. Psychol Aging.

[25] Cacioppo JT, Hawkley LC, Thisted RA (2010). Perceived social isolation makes me sad: 5-year cross-lagged analyses of loneliness and depressive symptomatology in the Chicago Health, Aging, and Social Relations Study. Psychol Aging.

[26] Hawkley L, Thisted R, Cacioppo J (2009). Loneliness predicts reduced physical activity: cross-sectional & longitudinal analyses. Health Psychol.

[27] Lin SZ (2015). Social exclusion and social inclusion: difficulties and solutions for returning to society of illicit drug users. Guizhou Soc Sci.

[28] Statistics New Zealand (2013). Loneliness in New Zealand: Findings from the 2010 NZ General Social Survey. (Wellington, New Zealand: Statistics New Zealand).

[29] Due TD, Sandholdt H, Waldorff FB (2017). Social relations and loneliness among older patients consulting their general practitioner. Dan Med J.

[30] Dahlberg L, Andersson L, McKee KJ, Lennartsson C (2015). Predictors of loneliness among older women and men in Sweden: A national longitudinal study. Aging Ment Health.

[31] Routasalo PE, Savikko N, Tilvis RS, Strandberg TE, Pitkala KH (2006). Social contacts and their relationship to loneliness among aged people - a population-based study. Gerontology.

[32] Ferreira-Alves J, Magalhaes P, Viola L, Simoes R (2014). Loneliness in middle and old age: demographics, perceived health, and social satisfaction as predictors. Arch Gerontol Geriatr.

[33] VanderWeele TJ, Jackson JW, Li S (2016). Causal inference and longitudinal data: a case study of religion and mental health. Soc Psychiatry Psychiatr Epidemiol.

[34] Zhong BL, Liu TB, Huang JX, Fung HH, Chan SS, Conwell Y, Chiu HF (2016). Acculturative Stress of Chinese Rural-To-Urban Migrant Workers: A Qualitative Study. PLoS One.

[35] Yu Z, Gu Y, Xiao S, Hu M, Zhou L (2017). Association between loneliness and risks of depressive episode among rural older people. J Cent South Univ (Med Sci).

[36] Cacioppo JT, Hughes ME, Waite LJ, Hawkley LC, Thisted RA (2006). Loneliness as a specific risk factor for depressive symptoms: cross-sectional and longitudinal analyses. Psychol Aging.

[37] Vogelsang E (2014). Self-rated health changes and oldest-old mortality. J Gerontol B Psychol Sci Soc Sci.

[38] Victor C, Grenade L, Boldy D (2005). Measuring loneliness in later life: a comparison of differing measures. Rev Clin Gerontol.

[39] Zhong BL, Liu TB, Chan SS, Jin D, Hu CY, Dai J, Chiu HF (2017). Common mental health problems in rural-to-urban migrant workers in Shenzhen. China: prevalence and risk factors. Epidemiol Psychiatr Sci.

